# Altered insulin-like growth factor-2 signaling is associated with psychopathology and cognitive deficits in patients with schizophrenia

**DOI:** 10.1371/journal.pone.0226688

**Published:** 2020-03-19

**Authors:** Yuan-Jian Yang, Tao Luo, Ying Zhao, Shu-Zhen Jiang, Jian-Wen Xiong, Jin-Qiong Zhan, Bin Yu, Kun Yan, Bo Wei

**Affiliations:** 1 Biological Psychiatry Laboratory, Jiangxi Mental Hospital/Affiliated Mental Hospital of Nanchang University, Nanchang, P.R. China; 2 Department of Psychiatry, Jiangxi Mental Hospital/Affiliated Mental Hospital of Nanchang University, Nanchang, P.R. China; 3 Department of Pharmacy, Union Hospital, Tongji Medical College, Huazhong University of Science and Technology, Wuhan, P.R. China; Nathan S Kline Institute, UNITED STATES

## Abstract

**Background:**

Schizophrenia is linked with abnormal brain neurodevelopment, on which IGF-2 (insulin-like growth factor-2) has a great impact. The purpose of this study was to assess the levels of serum IGF-2 and its binding proteins IGFBP-3 and IGFBP-7 in schizophrenia patients and the associations of these proteins with schizophrenia psychopathology and cognitive deficits.

**Methods:**

Thirty-two schizophrenia patients and 30 healthy controls were recruited. The PANSS and a neurocognitive test battery were used to assess schizophrenic symptomatology and cognition, respectively. Serum IGF-2, IGFBP-3 and IGFBP-7 levels were determined using ELISA.

**Results:**

The schizophrenia patients had a much lower content of serum IGF-2, IGFBP-3 and IGFBP-7 than controls. For the patients, IGF-2 levels were negatively correlated with the PANSS negative scores and positively associated with working memory, attention, and executive function. The correlations between IGF-2 and the PANSS negative scores, working memory or executive function were still significant after controlling for age, sex, education level, BMI, illness history and age of onset. No significant associations of IGFBP-3 or IGFBP-7 with the PANSS scores and cognitive function were observed in the patients.

**Conclusions:**

Our study demonstrates that serum IGF-2 was significantly correlated with negative and cognitive symptoms in patients with schizophrenia, suggesting that altered IGF-2 signaling may be implicated in the psychopathology and cognitive deficits in schizophrenia.

## Background

According to a hypothesis about schizophrenia, if an adult suffers from severe mental illness, the central nervous system must have developed in a disrupted manner [1,2]. This has been widely indicated by studies in areas of epidemiology, genetics and neuroimaging [3]. For example, structural brain abnormalities are apparent in the beginning stages of schizophrenia [4,5]; there were cases in which young people frequently suffered abnormal cognition and emotion abilities just before the appearance of the mental illness [6]; and primates suffering neonatal lesions had a disabled motion capability [7].

As a type of insulin-like protein, insulin-like growth factor (IGF) is an integral component of the cell system in human bodies that serves as a communication channel regarding the physiological circumstances [8]. Accumulated evidence has convincingly demonstrated that IGF has an indispensable and crucial role to play in nerve growth [9]. During the development of brains, IGF signaling regulates the growth survival, maturations, and proliferation of many types of nerve cells, such as astrocytes, oligodendrocytes, NPC (nerve precursor cell) and NSC (nerve stem cell) [9]. In addition, IGF sends basic signals to NSC cells directing them to grow into particular lineages in early development and influences their specific biological roles in late development, with the help of other nerve signals [9]. Individuals suffering from IGF gene mutations are vulnerable to physically disabled growth, nanocephaly or intellectual disability [10,11,12].

Two different IGF ligands, insulin-like growth factor-1 (IGF-1) and insulin-like growth factor-2 (IGF-2), have drawn much attention in recent studies. Researchers have shown that IGF-1 signaling plays a role in schizophrenia pathogenesis [13,14,15]. Specifically, plasma IGF-1 levels were decreased in antipsychotic-naive schizophrenia patients and were inversely correlated with positive symptom scores and hallucination subscores [13]. Antipsychotic treatment can increase serum IGF-1 levels in schizophrenia patients, and those patients with a greater increase showed a reduction in positive symptom scores to a higher extent [14]. Palomino et al. studied and showed that the negative symptoms of schizophrenia patients were correlated with their plasma IGF-1 level, regardless of whether they were in their first psychotic episode or one year later [15]. Furthermore, IGF-1 levels were also decreased in an animal model of schizophrenia [16]. In contrast to IGF-1, IGF-2 has not been as well characterized and is expressed in brains not only during the development period but also during adulthood [9]. According to recent studies in rodents, IGF-2 not only plays an important role in neurogenesis but also in cognition. The learning process increased brain IGF-2 levels, and exogenous administration of IGF-2 strengthened memory in mice [17,18]. IGF-2 levels were decreased in the hippocampus of old rats, and administration of IGF-2 rescued their aging-related memory loss [19]. In addition, overexpression of hippocampal IGF-2 could reverse memory and synaptic injuries in APP genetically modified mice via promoting the formation of dendrite spines and the transmission of excitatory synapses [20]. Moreover, a recent small genetic study showed that one genotype (ApaI) of the *IGF-2* gene, which is functionally associated with higher IGF-2, was associated with better selective attention performance in healthy individuals [21].

Recent reports have suggested that IGF-2 signaling is linked to schizophrenia. *IGF-2* was found to be the top downregulated gene in the prefrontal cortex of schizophrenia patients in a large CommonMind consortium RNA-sequencing study [22]. Prominent hypomethylation of an enhancer within the *IGF-2* gene was observed in isolated neurons from the prefrontal cortex of schizophrenia patients [23]. Akanji et al. assessed the levels of IGF-2 in male Arab patients with chronic schizophrenia, who had been receiving a stable dose of oral antipsychotic medications, showing that serum IGF-2 levels were significantly increased in schizophrenia patients and the levels of IGF-2 were positively correlated with atherogenic lipoproteins [24]. In addition, IGF-2 protein was downregulated in the hippocampus of mice lacking DiGeorge chromosome syndrome region 8 (Dgcr8), a candidate gene for 22q11.2 deletion-associated schizophrenia [25]. However, thus far, whether there is a relationship between IGF-2 signaling and the psychopathology and cognitive impairments in schizophrenia remains unknown. Therefore, this study aims to explore the potential function of IGF-2 signaling in the pathophysiology of schizophrenia by examining (1) whether serum IGF-2 was altered in Han Chinese patients who suffered from schizophrenia and (2) whether changes in IGF-2 levels were associated with the psychopathological symptoms as well as cognitive deficits of the patients. Given that the functions of IGF are modulated by IGF-binding proteins (IGFBPs), e.g., IGFBP-3 and IGFBP-7, this study also assessed changes in the levels of these binding proteins because they have been implicated in both neurodevelopment and cognitive function [18,26,27].

## Methods

### Schizophrenia patients and healthy controls

For this study, we recruited thirty-two schizophrenia patients who met DSM-IV criteria from Jiangxi Mental Hospital from Dec. 2016 to May 2017. Two psychiatrists confirmed their schizophrenia diagnosis. The exclusion criteria included the following: additional axis I DSM-IV diagnoses, axis II DSM-IV diagnoses, autoimmune, allergic, and neoplastic diseases, current pregnancy, and other physical disorders, such as cardiac block and cerebral infarction in the past 3 months. All the recruited patients were drug naive or had stopped taking any antipsychotics for at least 3 months when entering the study. For controls, we recruited at the same time thirty healthy people in nearby living communities with their sex, age, BMI (body mass index) and education level similar to the patients. We assigned a clinical psychiatrist to check the controls’ healthy condition, such as their current mental condition, personal mental disorder history, and family mental disorder history. The examination showed that all the controls had a good individual and familial psychiatric history.

All the patients and controls were of Han nationality. No participants suffered from substance dependence or substance abuse, and no participants were taking immunosuppressants. We carried out this research according to the requirements of Declaration of Helsinki, and our study received approval from the Institutional Review Board at Jiangxi Mental Hospital. We obtained consent from the patients and the controls or their legal guardians in written form. The capacity to consent for the participants in this study was determined with the University of California, San Diego Brief Assessment of Capacity to Consent (UBACC), which is a 10-item scale that includes questions focusing on understanding and appreciation of the information concerning the research protocol [28]. The authors in this study had access to information that could identify individual participants after data collection.

### Measurement of psychopathological symptoms and cognition

In our study, two psychiatrists were responsible for the measurement of the psychopathological symptoms for the patients by using the PANSS (Positive and Negative Syndrome Scale). Before the study, the two psychiatrists were simultaneously trained to use the PANSS. After that, they obtained an interobserver correlation coefficient of over 0.80 for the total scores of PANSS.

For the measurement of cognition, we used a set of neurocognition tests, the clinical dependability of which had already been verified and confirmed in population groups of Chinese people [29,30]. These tests consist of the following seven tasks:

TMT-A (Trail making test A): In this test, the participant is given a pencil and a piece of paper with numbered circles and asked to draw a line to connect the circles in the sequence of the numbers. The participant should draw the lines as quickly and accurately as possible, since the time taken for the drawing was used for grading.

BACS (Brief assessment of cognition in schizophrenia)-symbol coding task: In this test, the participant was given 133 pairs of digits and symbols and asked to copy the specific symbol as soon as its paired number was shown. This should be completely quickly because the number of symbols correctly finished within 120 seconds was used for the grading.

WMS-III spatial span (Wechsler memory scale-edition III-spatial span): In this test, the participant was faced with a board with ten cubes irregularly spaced on it. An administrator of this test first showed the participant combinations of the cubes in different ways and orders, forward and backward, and then asked the participant to recall the combinations. On each level of combinations, the participant was given two trials. The score was based on the number of recalled trials.

BVMT-R (Brief visual memory test-revised): In this test, the participant viewed six geometric figures, which were presented three times for ten seconds each time. Subsequently, the participant was asked to draw the figures on a piece of paper in the layout that they were presented in. The more figures that were correctly drawn, the better the score.

HVLT-R (Hopkins verbal learning test-revised): In this test, the participant was presented with twelve Chinese words, which were listed in three categories. The list was shown three times followed by a delay time of 25 to 30 minutes. Subsequently, the participant was asked to recall and speak out the words. Performance was graded in accordance with the number of words correctly recalled.

CPT-IP (Continuous performance test-identical pair): In this test, the participant saw digital numbers of 2, 3 and 4 digits flashing on a computer screen. The participant was asked to immediately click the mouse after the same number was repeatedly flashed on the screen. In this test, the participants were expected to identify the appropriate target 90 times, miss a target (i.e., trigger a false alarm) 90 times, and randomly respond approximately 270 times.

SCWT (Stroop color-word test): In this test, the participant was presented with three pages: first, a word page, on which color words are written in black; second, a color page, on which several rows of Xs were written in different colors; and third, a word-color page, on which the same color words on the first page were written in the colors from the second page, although they are not the same color as the color that the written word spells. In each trial, the participant was presented with 100 words and asked to read them as quickly as possible in 45 seconds. The number of correctly read words was used for grading.

For the above seven tests, we grouped them into six cognitive domains, i.e., executive function, attention, verbal learning, visual memory, working memory, and processing speed, which respectively cover the Stroop color-word test, CPT-IP, HVLT-R, BVMT-R, WMS-III spatial span, and TMT-A and BACS-symbol coding.

### Measurement of IGF-2, IGFBP-3 and IGFBP-7

Antecubital venous blood was sampled in the morning from seven to nine after overnight fasting. After that, we separated the serum, aliquoted the samples and immediately stored them at -80°C.

We measured the levels of serum IGF-2 (Catalog # SEA051Hu), IGFBP-3 (Catalog # SEA054Hu), and IGFBP-7 (Catalog # SEB673Hu) using ELISA with available kits on the market (Wuhan USCN Business, Wuhan, China). The sensitivities for IGF-2, IGFBP-3 and IGFBP-7 were 0.264, 0.071 and 0.057 ng/ml, respectively, with variation coefficients of inter- and intra-assay of 10%, 12%, respectively. In briefly, the same volume of standard or sample was added to the appropriate microplate well with a biotin-conjugated antibody specific to IGF-2, IGFBP-3 or IGFBP-7. Next, Avidin conjugated to Horseradish Peroxidase (HRP) was added to each microplate well and incubated. After TMB substrate solution was added, only those wells that contain IGF-2, IGFBP-3 or IGFBP-7, biotin-conjugated antibody and enzyme-conjugated Avidin would exhibit a change in color. The enzyme-substrate reaction was terminated by the addition of sulphuric acid solution and the color change was measured spectrophotometrically at a wavelength of 450 nm. Each sample was measured in duplicate. We averaged the duplicate readings for each standard, control, and sample, subtracted the average zero standard optical density (O.D.), and then constructed a standard curve by plotting the mean O.D. and concentration for each standard and drew a best fit curve through the points on the graph. The concentration of IGF-2, IGFBP-3 or IGFBP-7 in the sample was then determined by comparing the O.D. of the sample to the standard curve. We assigned the same investigator, who was blind to the clinical situation, to assay all the samples. To prevent inter-assay variance, we analyzed all the samples in the same assay.

### Statistical analysis

Chi-square tests were used for categoric variables, and Student's *t*-test or analysis of variance (ANOVA) was used for the continuous variables for the comparison of the demographic and clinical variables between the patients and the controls. Since the IGF-2, IGFBP-3 and IGFBP-7 data were normally distributed in the healthy controls and patients (*p* > 0.05, Kolmogorov-Smirnov 1-sample test), we used one-way ANOVA to compare these protein levels between the two groups. We performed ANCOVA (analysis of covariance) if a crucial difference existed in the two groups to verify the influence of sex, age, education level, and BMI. For the assessment of the relationships among those factors, we used Pearson's product moment correlation ratios. For the assessment of the relationships among serum IGF-2, IGFBP-3, or IGFBP-7 levels and psychotic and cognitive symptoms controlling for clinical variables, such as sex, age, education level, BMI, illness history, and onset age, we used partial correlation analysis in this study. We present the data from the analysis as the mean ± SD, and we adopted two-tailed significance values of 0.05.

For the calculation of cognitive scores, we first converted all the test scores into standardized z scores. For this purpose, we set the sample mean value in every measurement to zero and at the same time set the standard deviation to one. In the cognition areas with two tests, we determined summary scores by first calculating the average value of the z scores in the two tests and then converting the average value to a z score with an average value of zero and a standard deviation value of one.

## Results

### Demographic data and cognition

The demographic data of both the healthy controls and the schizophrenia patients are reported in Table 1. The two groups were not significantly different in terms of sex, age, education level or BMI (all *p* > 0.05). We present the results of the cognition tests in Table 2 for the patients with schizophrenia and the healthy controls. The findings indicated that the schizophrenia patients performed worse than the healthy controls (all *p* < 0.05) on all the cognition tests. With the exception of the BVMT-R score (*p* = 0.063), the two groups were still very different even after we adjusted for sex, age, education level, and BMI.

**Table 1:** Demographics of patients and healthy control subjects.

	Patients with schizophrenia (n = 32)	Control subjects (n = 30)	*F* or *X*^2^	*p*
Age (years)	30.0 ± 8.5	33.5 ± 9.1	2.458	0.122
Sex, M/F	17/16	14/16	0.148	0.701
Education (years)	11.6 ± 5.0	12.6 ± 4.5	0.571	0.453
BMI (kg/m^2^)	21.7 ± 2.3	21.2 ± 1.7	0.954	0.333
Age of onset (years)	23.3 ± 5.9	–	–	–
Duration of illness (years)	5.9 ± 5.4	–	–	–
PANSS total score	80.8 ± 11.1	–	–	–
Positive subscale	22.6 ± 4.1	–	–	–
Negative subscale	13.8 ± 4.5	–	–	–
General psychopathology subscale	44.5 ± 6.0	–	–	–

BMI = body mass index; PANSS = Positive and Negative Syndrome Scale.

**Table 2:** Comparison of cognitive function between the two groups.

Cognitive tests	Healthy controls (n = 30)	Patients with schizophrenia (n = 32)	*F*	*p*	*Adjusted F*	*p*
TMT-A	42.3 ± 9.8	72.3 ± 28.9	31.375	<0.001	6.627	<0.001
BACS-SC	66.0 ± 6.3	35.3 ± 14.3	115.986	<0.001	24.709	<0.001
WMS-III-SS	17.0 ± 2.2	14.6 ± 3.2	11.985	0.010	3.979	0.004
HVLT-R	27.3 ± 4.6	19.6 ± 6.0	31.407	<0.001	6.845	<0.001
BVMT-R	29.0 ± 10.2	21.3 ± 4.5	4.427	0.040	2.233	0.063
CPT-IP	3.25 ± 1.1	1.4 ± 0.8	54.803	<0.001	11.128	<0.001
Stroop color-word test						
Word raw score	82.3 ± 9.4	54.8 ± 15.3	88.271	<0.001	17.849	<0.001
Color raw score	51.8 ± 9.0	36.6 ± 14.7	23.902	<0.001	4.837	0.001
Color-word raw score	37.8 ± 7.1	21.4 ± 12.7	38.286	<0.001	7.883	<0.001

TMT-A = trail making test, part A; BACS-SC = brief assessment of cognition in schizophrenia-symbol coding; WMS-III-SS = Wechsler memory scale-3rd edition-spatial span; HVLT-R = Hopkins verbal learning test-revised; BVMT-R = brief visual-spatial memory test-revised; CPT-IP = continuous performance test-identical pairs. Adjusted *F* indicates the *F* value controlled for age, sex, years of education, and BMI.

### The levels of serum IGF-2, IGFBP-3 and IGFBP-7 in schizophrenia patients and healthy controls

**Fig 1:**
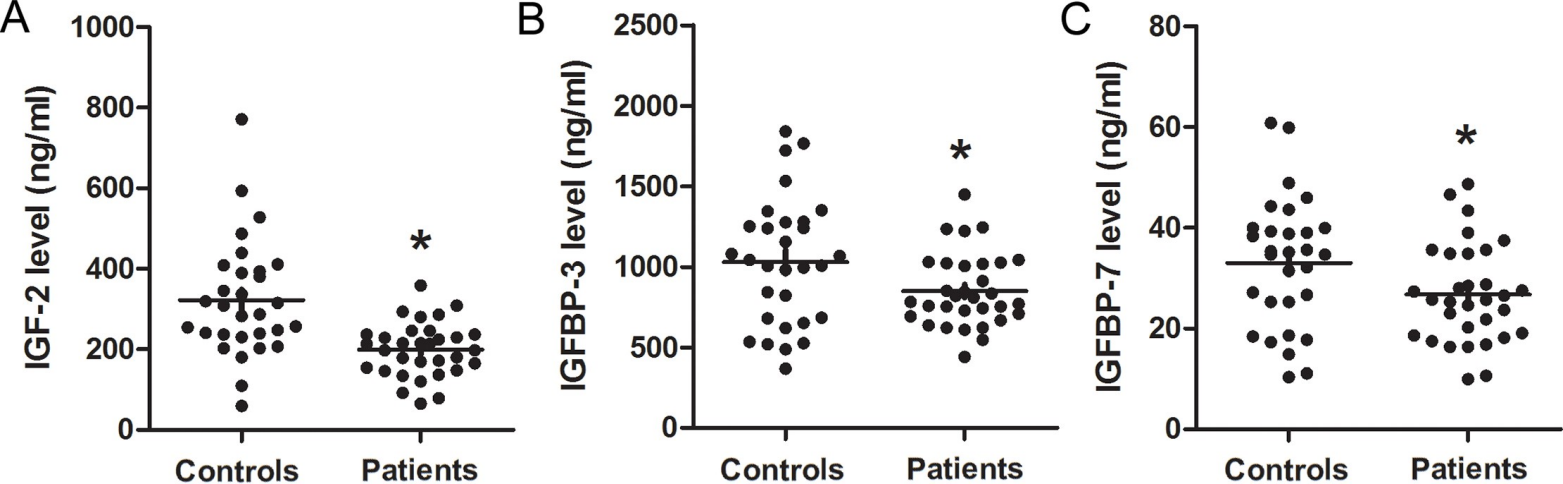
This figure presents a scattergram of serum IGF-2 (A), IGFBP-3 (B) and IGFBP-7 (C) levels from patients with schizophrenia (n = 32) and from control subjects (n = 30). The sample means are indicated by black bars. *p < 0.05.

The levels of these factors in both the healthy controls and the schizophrenia patients are shown in Table 3 and Fig 1. The patients had much lower levels of IGF-2, IGFBP-3, and IGFBP-7 than the controls (all *p* < 0.05). This was still observed even after we added sex, age, education level, and BMI to the ANOVA as covariates and examined the effects of these factors: IGF-2 (*F* = 2.698, *p* = 0.040), IGFBP-3 (*F* = 2.547, *p* = 0.049), and IGFBP-7 (*F* = 2.664, *p* = 0.041). The serum levels of these proteins in males and females in both groups were similar (*p* > 0.05). Furthermore, no correlations existed among the demographic parameters and the IGF-2, IGFBP-3 or IGFBP-7 levels for both the patients and the controls (all *p* > 0.05).

**Table 3:** Serum IGF-2, IGFBP-3 and IGFBP-7 levels in the patient and control groups.

	Healthy controls (n = 30)	Patients with schizophrenia (n = 32)	*F*	*p*	*Adjusted F*	*p*
IGF-2 (ng/ml)	355.7 ± 70.4	199.2 ± 67.2	10.051	0.002	2.698	0.040
IGFBP-3 (ng/ml)	1034.5 ± 390.7	849.7 ± 227.1	5.633	0.025	2.547	0.049
IGFBP-7 (ng/ml)	33.0 ± 12.9	26.8 ± 9.8	4.670	0.035	2.664	0.041

Adjusted *F* indicates the *F* value controlled for age, sex, education, and BMI.

### Association of serum IGF-2, IGFBP-3, and IGFBP-7 levels with psychopathological symptoms in the schizophrenia patients

For the schizophrenia patients, the correlation analysis with serum IGF-2 levels revealed a significant association between IGF-2 levels and the PANSS negative scores (*r* = -0.452, *p* = 0.009), but no significant associations between serum IGF-2 levels and PANSS total scores, positive scores or general scores (all *p* > 0.05) (Table 4). Partial correlation analysis indicated that serum IGF-2 had a significant correlation with PANSS negative scores even after controlling for sex, age, education level, BMI, illness history and onset age (*r* = -0.408, *p* = 0.035) (Table 4). However, the correlation analysis with IGFBP-3 or IGFBP-7 showed that there was no significant association of either IGFBP-3 or IGFBP-7 levels with the PANSS total or subscale scores in schizophrenia patients (all *p* > 0.05).

**Table 4:** Correlations between serum IGF-2 levels and psychopathology and cognitive function in patients.

	IGF-2 levels			
	Pearson's correlation		Partial correlation analysis	
**Psychopathology**	*r*	*p*	*r*	*p*
PANSS total scores	0.019	0.919	0.141	0.683
PANSS positive subscores	0.042	0.820	0.045	0.823
PANSS negative subscores	-0.452	0.009	-0.408	0.035
PANSS general subscores	0.220	0.225	0.387	0.046
**Cognitive function**				
processing speed	0.158	0.389	0.059	0.768
working memory	0.546	0.001	0.515	0.006
visual memory	-0.198	0.277	0.049	0.810
verbal learning	-0.002	0.990	-0.153	0.447
attention	0.405	0.021	0.352	0.071
executive function	0.606	<0.001	0.543	0.003

### Correlation between IGF-2, IGFBP-3, IGFBP-7 levels and schizophrenia cognition symptoms

We grouped the cognitive tests into six cognitive areas, i.e., executive function, attention, verbal learning, visual memory, working memory, and processing speed, which respectively cover the Stroop color-word test, CPT-IP, HVLT-R, BVMT-R, WMS-III spatial span, and TMT-A and BACS symbol coding. We tested the correlations between serum IGF-2, IGFBP-3, or IGFBP-7 levels and cognitive performance for both the patients and controls.

The correlation analyses revealed a crucial positive association of serum IGF-2 levels with the working memory index (*r* = 0.546, *p* = 0.001), the attention index (*r* = 0.405, *p* = 0.021), and the executive function index (*r* = 0.606, *p* < 0.001) in the schizophrenia patients (Table 4). Serum IGF-2 levels were not correlated with the processing speed index (*r* = 0.158), the visual memory index (*r* = -0.002) or the verbal learning index (*r* = -0.198) (all *p* > 0.05). Partial correlation analysis indicated that serum IGF-2 levels still had a significant correlation with working memory (*r* = 0.515, *p* = 0.006) and executive function (*r* = 0.543, *p* = 0.003), even after controlling for sex, age, education level, BMI, illness history and onset age (Table 4). However, there was no significant association of either serum IGFBP-3 or IGFBP-7 levels with the cognitive test scores in the patients (all *p* > 0.05).

## Discussion

The main findings in this study are as follows: (1) Serum IGF-2, IGFBP-3, and IGFBP-7 levels were significantly lower in the Chinese schizophrenia patients than the controls. (2) Serum IGF-2 levels had a negative correlation with PANSS negative symptoms and a significant positive association with working memory and executive function in patients. (3) Neither serum IGFBP-3 nor IGFBP-7 levels had a significant correlation with the psychotic and cognitive symptoms in the patients.

The IGF system is mainly composed of IGF-1 and IGF-2, the binding proteins (IGFBPs) and the cell-surface receptors. Relative to IGF-1, little is known regarding IGF-2, which is expressed in brains during fetal development and is also the most highly expressed IGF in the central nervous system of adults [31]. In biological fluids, IGF-2 usually binds with IGFBPs, which lengthen its half-life and regulate its usability and biological activity [8]. Among the IGFBPs, IGFBP-3 and IGFBP-7 have been shown to be implicated in neurodevelopment and cognition [18,26,32]. Our paper indicates that there were much lower levels of serum IGF-2, IGFBP-3 and IGFBP-7 in Chinese schizophrenia patients. After controlling for sex, age, and BMI, the content of these proteins still differed between the two groups. However, in contrast to our findings, one previous study performed in male Arab subjects demonstrated that serum IGF-2 content was much higher in schizophrenia patients, and no change was found in IGFBP-3 levels [24]. A possible explanation of the discrepancies in serum IGF-2 and IGFBP-3 levels may be different ethnicities (Chinese *vs*. Arab). Indeed, ethnicity impacts IGF-2 and IGFBP-3 levels in the serum of normal healthy subjects [33]. Besides, the effect of antipsychotic medications could also be a contributor to these discrepancies (stable antipsychotic medication *vs*. medication-free). Nevertheless, although there was an inconsistency, a change in serum IGF-2 content in schizophrenia patients was found in both studies. In light of evidence that IGF-2 is a potent neural growth-promoting factor and that adult-onset disorders could trace back to development [2,9], the results indicate that IGF-2 signaling might be involved in schizophrenia pathophysiology. However, whether a change of IGF-2, IGFBP-3 and IGFBP-7 in patients is caused by schizophrenia itself or other confounding factors, for example, influenced by antipsychotic drugs, is still unknown. In this study, we recruited schizophrenia patients who were not taking any antipsychotic medication for at least three consecutive months before participating in this study. Thus, we postulate that the alterations in IGF-2 signaling in schizophrenia patients is more likely to be related to the illness per se, rather than a phenomenon secondary to medication treatment. However, future studies are required to test this hypothesis by measuring IGF-2 levels in drug-naive first-episode schizophrenia patients.

This study reveals that serum IGF-2 levels had an inverse correlation with the PANSS negative scores, and this correlation remained significant after controlling for sex, age, education level, BMI, illness history, and onset age, suggesting that patients with lower IGF-2 levels were more prone to serious negative symptoms. The severity of psychotic symptoms has been shown to be associated with structural alterations in the cortex and striatum [34]. IGF-2 could promote synapse development, spine maturation, and memory formation in the brain [18,35]. Pai et al. showed that the methylation level of an enhancer within the *IGF-2* gene, which targets the nearby tyrosine hydroxylase (*TH*) gene responsible for dopamine synthesis, was reduced in the frontal cortex of schizophrenia patients [23]. Deletion of an intergenic *IGF-2* enhancer in mice led to a decrease in TH protein levels and in dopamine in the striatum [23]. In addition, pathway enrichment analysis identified that *IGF-2* enhancer deletion would result in alterations in cell proliferation/development, protein synthesis, immune responses, neurodevelopment, and cytoskeletal remodeling in the frontal cortex and striatum [23]. Therefore, we postulate that decreased IGF-2 in schizophrenia might cause structural alterations and dopamine dysfunction in the cortex and striatum to aggravate the negative symptoms. However, this explanation is quite speculative. Further research using animal experiments is needed to address this assumption.

Disrupted cognition is a main characteristic of schizophrenia [3]. A variety of cognitive domains, such as processing speed, attention, working memory, visual memory, verbal learning, and executive function, have been reported to be impaired in schizophrenia. Our present study shows that schizophrenia patients displayed poorer performance in processing speed, working memory, attention, visual memory, and executive function than the normal controls, identical to previous studies [30,36]. IGF-2 has been strongly linked to cognition in the last decades [37]. Specifically, the ApaI polymorphism of the *IGF-2* gene was associated with the general cognition and selective attention index in healthy individuals [21]. A link was found between DNA methylation levels of *IGFBP-1* gene and adult working memory performance in a monozygotic twin sample [38]. Both serum IGF-2 and IGFBP-3 levels were shown to be correlated with normal age-related cognitive decline in the Caerphilly Prospective Study from a cohort of 746 men [39]. Furthermore, injection of recombinant IGF-2 into the hippocampus enormously improved memory retention and reduced forgetting in rats [17,18]. In this study, we found that serum IGF-2 levels were positively correlated with working memory and the executive function of schizophrenia patients. Given that IGF-2 is abundantly expressed in brain regions that are relevant to working memory and executive function, like hippocampus and prefrontal cortex [18,40,41], these findings demonstrate that abnormal IGF-2 signaling is implicated in cognitive impairments of schizophrenia. IGF-2 could affect cognition via many pathways [18,25,42]. For example, administering IGF-2 in rats enhances memory retention and prevents forgetting via promoting new protein synthesis, the activation of glycogen-synthase kinase 3 and the expression of AMPA receptor in hippocampus [18]. IGF-2 treatment improved cognitive/executive functions by targeting the AMPK-mTOR-S6K pathway in a mouse model of Autism [42]. Exogenous IGF-2 could reverse the spatial working memory deficits in Dgcr8(+/-) mice, a neurodevelopmental defect model for schizophrenia, by rescuing the proliferation of adult neural stem cells in the hippocampus [25]. However, further studies are still needed to elucidate the mechanisms of IGF-2 in the regulation of cognition in schizophrenia.

The availability and bioactivity of IGF-2 are modulated by IGFBPs in biological fluids [8]. Numerous studies have demonstrated that IGFBP-3 and IGFBP-7 are implicated in neurodevelopment and cognitive function [18,26,32]. Circulating IGFBP-3 levels were markedly decreased in AD patients and normal aging individuals, and the levels of IGFBP-3 were associated with cognitive status in these populations [43,44]. IGFBP-7 plays an important controlling role in memory consolidation [32]. Changes in IGFBP-7 levels have been correlated with postoperative cognitive dysfunction and AD-like memory impairments [27,45]. Our present study demonstrated that the levels of both serum IGFBP-3 and IGFBP-7 were markedly reduced in schizophrenia patients, while there was no significant association between either IGFBP-3 or IGFBP-7 levels and the psychotic and cognitive symptoms in the patients. These results indicated that a low level of serum IGFBP-3 and IGFBP-7 is probably a phenomenon secondary to decreased IGF-2 protein, rather than related to illness per se. However, this failure to observe a relationship might also have come from a lack of statistical power since there were not many patients and controls in this study. Further study in larger patient samples is needed to address these possibilities.

This study has the following limitations. First, although we found close associations between serum IGF-2 and the psychotic and cognitive symptoms in schizophrenia patients, the mechanism through which IGF-2 affects schizophrenia-related behaviors is still unknown. Further research using animal experiments is needed to reveal the mechanisms of IGF-2 signaling in schizophrenia. Second, only serum IGF-2 content was considered in this paper, but its content in cerebral spinal fluid was not assessed. Whether peripheral IGF-2 reflects similar changes in the central nervous system is not known. Third, the IGF system is affected by nutritional status [46]. Prealbumin and albumin, indicators of nutrition conditions, should also be assessed in the analysis of patients and controls. In addition, there were not many patients and controls in this study, and further study in larger patient samples is needed.

## Conclusions

This study indicated that serum IGF-2, IGFBP-3 and IGFBP-7 levels of Han Chinese schizophrenia patients were decreased and serum IGF-2 levels were associated with both negative and cognitive symptoms of the patients, indicating that altered IGF-2 signaling probably contributes to the psychopathology and cognitive impairments of schizophrenia. Nevertheless, to understand the mechanism of altered IGF-2 signaling in schizophrenia patients, more studies are necessary, which could not only help to essentially understand schizophrenia but also provide new methods for the treatment of schizophrenia.

## Supporting information

S1 Data (ZIP)
